# Effectiveness of the population health and environment approach in improving family planning outcomes in the Gurage, Zone South Ethiopia

**DOI:** 10.1186/s12889-015-2484-9

**Published:** 2015-11-13

**Authors:** Makeda Sinaga, Ahmed Mohammed, Negash Teklu, Kristen Stelljes, Tefera Belachew

**Affiliations:** Nursing Department, College of Public Health and Medical Sciences, Jimma University, Jimma, Ethiopia; PHE Ethiopia Consortium, Addis Ababa, Ethiopia; Population and family health Department, College of Public Health and Medical Sciences, Jimma University, Jimma, Ethiopia

**Keywords:** Population, Health, Environment, Family planning, Integration, Gurage Zone

## Abstract

**Background:**

Family planning is a strategy of balancing population growth with economic development for sustainable use of natural resources. A high population growth induces increased demand for resources and the rate at which these resources are exploited. Population, health and environment are connected inextricably. Population growth unbalanced with economic development leads to food insecurity which exposes households to the consumption of food with reduced quality and quantity leading to increased risk of malnutrition and poor health. Food insecurity again obliges people to encroach into the natural environment leading to a spiraling progress to destitution. A study in the Philippines provided concrete evidence that integrated development programming incorporating population, health, and the environment (PHE) can be more effective in lowering population growth rates and preserving critical coastal ecosystems than single-sector development interventions”. Although the PHE approach has been implemented for 5 years (2008**–**2012) Guraghe Zone of South Ethiopia, its outcomes have not been evaluated. The objective of this study was to evaluate the effectiveness of PHE approach for achieving family planning (FP) outcomes in Gurage Zone.

**Methods:**

A comparative cross-sectional study was conducted in October, 2012. A total of 962 married women in the reproductive age group were included in the study. Data were collected using an interviewer administered Amharic version questionnaire. Descriptive statistics and multivariable logistic regression analyses were performed to compare the PHE and non-PHE Woredas (district) based on family planning parameters adopted from Measure Evaluation Manual.

**Results:**

Comparison of non-new family panning acceptor women showed that PHE Woreda had a significantly high CPR compared to non-PHE (78 % vs 52 %, *P < 0.0001*). Among these sub-groups, women in the PHE Woreda were over four times more likely to use family planning methods during the study period (*P < 0.0001*) compared with women in the non-PHE Woreda. Women whose husbands’ supported their use of family planning methods were 17 times as likely to use family panning methods (AOR: 17.2, 95 % CI [11.1, 26.8]), *P* < 0.0001. This was even increased to 20 times more when we did sub-group analysis only for women who were not new acceptors (AOR: 20.4:95 % CI [9.7, 42.7]), *P* < 0.0001. The qualitative results showed that there was a better integration of FP, health and environmental issues into the grassroots level interventions in the PHE Woreda through using students as a medium for reaching parents on family planning and environmental issues.

**Conclusions:**

The findings suggest that overall; PHE has positive outcomes in FP behaviors both among married women and their husbands. Integration of population, health and environmental issues need to be strengthened and scaled up to sustain the positive FP behaviors such as support of FP use. Strategies used in the PHE Woredas such as using schools and students as medium for integrated PHE interventions are commendable approaches that need to be strengthened.

## Background

Family planning (FP) is defined as the ability of individuals and couples to anticipate and attain their desired number of children and the spacing and timing of their births [1]. The major purpose of FP at macro-level is balancing population growth with carrying capacity of the environment. Experience over the last couple of decades in Ethiopia has shown that as human population increased, the population carrying capacity of the environment decreased. A high population growth rate induces increased demand for resources and the rate at which these resources are exploited. Population and environment are connected inextricably [2]. In Ethiopia, environmentally harmful and economically counterproductive methods of exploiting land and associated resources (forests, animal resources, etc.) are resorted to in order to meet immediate needs. As a consequence of this, climatic conditions are becoming erratic and soil quality is declining at an alarming rate. Therefore, FP intervention is an environmental imperative to respond to this subtle interplay between population growth, environmental degradation and poor nutrition and health leading to a spiraling race to poverty. However, in less developed countries including Ethiopia, there is limited access to birth control, as well as cultural practices encourage women to stay home and have babies, leading to rapid population growth.

Results from the 2011 Ethiopian Demographic and Health Survey showed a good progress towards increasing FP coverage nationwide with an increase of CPR among married women to 27 % from 15 % CPR in 2005 [3]. However, this improvement is still far from the ambitious goal set by the Ethiopian government to achieve a CPR of 65 % by 2015. With an annual population growth rate of with 2.6 %, the population of Ethiopia will double after 27 years [4].

To address the current low rate of FP outcomes in Ethiopia, population, health and environment (PHE) offers a step in the right direction in a flexible and innovative way to keep pace with today’s rapidly changing world and lays the foundation for empowering the generations to come [5]. In 2009, PHE Ethiopia consortium developed the following definition of PHE: “holistic, participatory development approach whereby issues of environment, health and population are addressed in an integrated manner for improved livelihoods and sustainable well-being of people and ecosystems.” In general, unlike the vertical FP services used hitherto, PHE initiatives aim to address the complex connection between humans, their health and the environment in providing FP services.

PHE interventions are a coordinated and integrated set of activities that include goals and interventions in the PHE sectors. Evidence from around the world demonstrates that the PHE approach is the way forwards for sustainable solutions to population and environmental problems. A study in the Philippines [6] demonstrated that an integrated development programming incorporating population, health, and the environment (PHE) was effective in lowering population growth rates and preserving the environment compared with single-sector development interventions. It has also been shown that an integrated approach to conservation and reproductive health generates higher impacts on human and ecosystem and on health outcomes compared to the independent vertical interventions [6-7]. Better reproductive health outcomes including increased contraceptive access and a significant decrease in the average number of children born to women and trends showing a significant reduction in income-poverty among young adults and added values has been reported [6, 8]. It is believed that the PHE approach has lead to an increase in access to FP and reproductive health services in remote communities in Madagascar, Kenya, Southern Asian coral triangle and Philippines [8]. Experiences from Madagascar and Philippines showed that the PHE approach of integrating the environment into reproductive health and FP programs not only integrated health into natural resource management projects prompting greater participation of women and adolescent girls, but also encouraged men and adolescent boys to get involved [8-9]. The integrated PHE approach leads to a more effective and sustainable solution to the population and environmental issues through empowering women and ensuring their involvement in the conservation activities [10] and through the economies of scale that the strategy ensures [6]. For instance; in the Philippines, an integrated approach improved both reproductive health and coastal resource management more than single-sector programs strongly suggesting that the integrated approach adds value [6]. The PHE approach also helps build trust with community as it aims at their priority issues including health services offering a fertile entry point for fostering community dialogue and concerted effort at the grassroots level.

It has been demonstrated that the integrated approach makes the PHE approach more effective and sustainable than delivering these services in stand-alone or parallel programs [6-7]. PHE projects in Ethiopia have been designed by local organizations and communities to identify goals that cannot be met without addressing basic needs and rights. For instance, Gurage People Self Development Organization (GPSDO) has incorporated agriculture extension and soil conservation into its family planning (FP) work to improve both food security and the health of women and children in the zone. As PHE interventions are relatively new in Ethiopia, there is a need to determine the effectiveness of the Ethiopian PHE model for achieving FP goals. This study aims to compare FP outcomes between PHE Woredas and non-PHE Woredas of the Gurage Zone.

## Methods

### Study area and setting

**Fig. 1:**
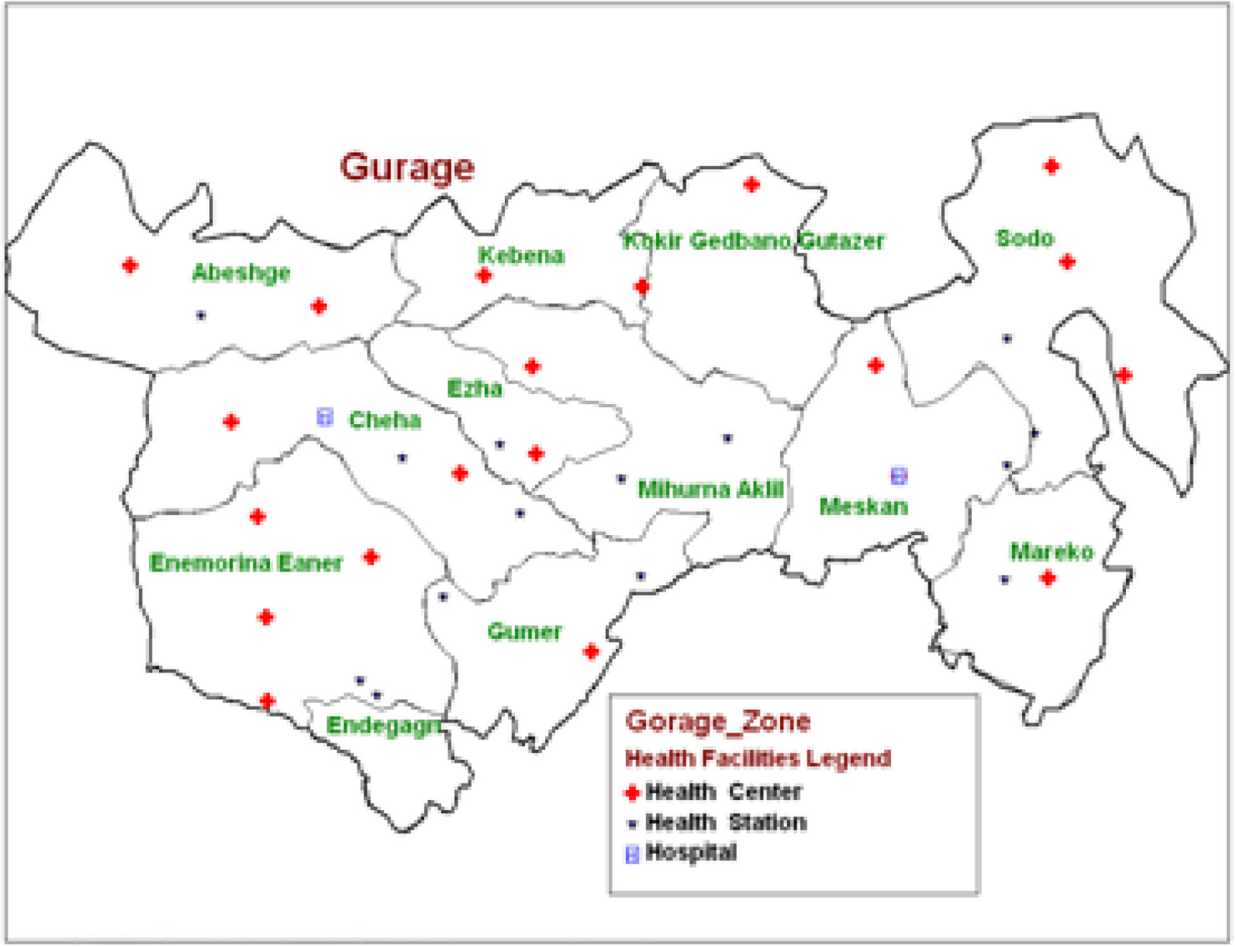
Map of Guraghe zone

The Gurage zone is located in the central and southeastern mountainous area of Ethiopia in the Southern Nations, Nationalities and Peoples Region (SNNPR). It consists of 13 Woredas (Fig. 1), of which, GPSDO works in nine Woredas involving 250 kebeles (The smallest political administrative units). It is implementing an integrated package of environmental protection, livelihood improvement, health and education interventions (PHE integration) in 45 kebeles located within four Woredas: Cheha, Getta and Mehur-Aklil. The population of the zone is 1.4 million, of which 90 % live in rural areas. The Gurage Zone is one of the most densely populated zones in Ethiopia, with the maximum density reaching 441 persons per square kilometer. The national average for Ethiopia is 281 persons per square kilometer, while the average for SNNPR is 106 persons per square kilometer. The primary occupation in the zone is subsistence farming. As a result of the high population density and traditional farming practices, there is extensive soil erosion in the area. Crop yields have also declined as the quality of the soil has declined due to overuse. This environmental degradation contributes to food insecurity in the zone. Few families own livestock and most farm plots are less than one acre. According to the zonal agricultural department, only 10 % of families have at least one complete meal per day. Many husbands also migrate to the cities and have business there and come back to their rural wives on the Meskel Holiday, which is celebrated in September. The Woreda health offices use the holiday an opportunity to distribute family planning services to prevent unwanted pregnancies.

### Study design and sampling techniques

A comparative cross-sectional study was carried out during October, 2012 to determine the FP outcomes in PHE and non-PHE Woredas in Gurage Zone. Detailed background data were collected from married couples using structured questionnaires. The study involved a total of 960 married women of in the reproductive age (15***–***49 years old) selected using a simple random sampling technique stratified by the PHE and non-PHE areas. The sample was further allocated to kebeles in each of the two Woredas in the PHE (Mihur Aklil) and non-PHE (Gumer) Woredas. Epinfo stat calculator was used to determine the sample size for the study using a formula for the estimation of comparative cross-sectional study with the following assumptions:

$$ n=\frac{{\left(Z\frac{\alpha }{2}\sqrt{\left(1+\frac{1}{r}\right)P\left(1-P\right)}+Z\frac{\beta }{2}\sqrt{P1\left(1-P1\right)+\frac{P2\left(1-P2\right)}{r}}\right)}^2}{{\left(P1-P2\right)}^2}\times 2\left(\mathrm{design}\ \mathrm{effect}\right) $$

Power (1- ß) =95 %

Zα/_2=_Standard normal variable at 95 % confidence level (1.96)

P1 = Expected CPR in non-PHE Woredas (P1) =33.46 % [11].

P2 = 43 % with an assumed odds ratio of 1.5,

R = Ratio of sample population from PHE to non-PHE Woredas (1:1).

This gives a minimum sample size of 480 married women. Multiplying this by a design effect of 2 for the cluster sampling gives a final sample size of 960 married women.

For the in-depth interview 22 key informants pooled from different stakeholders were purposively selected from GPSDO staff, Health Extension Workers (HEWs), development agents (DAs), Community Based Reproductive Health Agents (CBRHAs), Woreda officials from relevant sectors such as health, agriculture, forestry workers education and students both in PHE and non-PHE Woredas.

**Fig. 2:**
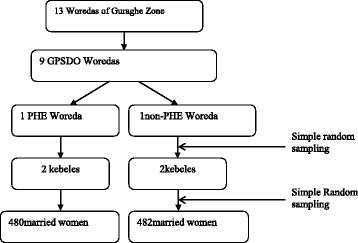
Sampling framework

Within each Woreda, kebeles were randomly selected as community survey sites. To the extent possible, the Woredas and kebeles were matched with similar demographic characteristics to make a fair comparison across PHE and non-PHE sites. The total sample from the quantitative study was allocated to the different Woredas in the PHE and non-PHE areas proportional to the size of their population. Within each kebele, the study subjects (married women 15–49 years) were selected using simple random sampling technique (Fig. 2).

### Measurement

The study generated data used to compare FP outcomes in PHE and non-PHE Woredas. Emphasis was given to indicators that are used for the GPSDO project outcomes which include indicators taken from the MEASURE Evaluation family planning and reproductive health indicators [12].

Variables including: contraceptive prevalence rate (CPR), percent of women of reproductive age who have heard of at least three methods of FP, percent of the population who knew at least one source of modern FP services or supplies, number of new acceptors to modern contraceptives (women who used modern contraceptive methods for the first time in their life during the last 1 year), percent of men who support the use of modern contraception for themselves or their partners were captured as measures of family planning outcomes.

The questionnaires focused on knowledge, and practices related to FP to determine if there is a difference in FP outcomes between the PHE and the comparison vertical FP sites. Questions in the survey were based on items adapted from other surveys such as DHS.

A survey questionnaire was developed and administered by data collectors after pre-test and necessary modifications were made. The data collectors and supervisors were trained on data collection instruments, procedures and study objectives and briefed on any changes made to the survey tool before they began data collection. For the actual survey, households were selected randomly using the list of households in kebele administration as a sampling frame. Within each household, married women of reproductive age were interviewed. Interviews were conducted by trained interviewers fluent in the local language (Guragigna).

Both woreda level and community level key informants were interviewed on the modalities of integration of family planning with conservation activities using semi-structured interview guide. All activities were conducted under close supervision of PHE Ethiopia Consortium staff in order to ensure quality and appropriateness as well as to build the capacity of PHE Ethiopia consortium staff in conducting high quality research projects.

### Operational definitions

#### PHE-Woreda

The PHE Woreda is Mihurna Aklil Woreda where family planning services are given integrated with environmental conservation activities using both community level volunteers, campaign and in schools and using students as a medium to educate their parents on population health and environment.

#### Non-PHE Woreda

Non- PHE Woreda (Gummer) is the woredaoreda where, family planning services are delivered as separate services vertically with no much emphasis on the integration with the environment and health.

### Data analysis

Data from the community survey were analyzed using SPSS for windows version 16.0. First descriptive statistics were provided using means and proportions. Bivariate logistic and linear regressions were performed to select variables that are candidates for entry into the adjusted model. Multivariable linear regression model was fitted to identify variables that have an independent effect on FP knowledge. A second multivariable logistic regression was performed to identify independent predictors of current FP use. In order to avoid the confounding effect of the Meskel Holiday family planning campaigns, logistic regression model was fitted for non-new acceptors of family planning. Statistical significance was declared at *P* < 0.05.

Qualitative data obtained from in-depth interview was grouped under themes; color coded and analyzed using thematic frameworks. The results were presented in triangulation with the quantitative results using direct quotes as illustrations.

### Ethical considerations

Verbal consent of the study participants was requested before data collection. Permission was granted from the Gurage Zonal and respective woreda health offices to conduct the study in the respective woreda. The study protocol was ethically reviewed and approved by the Jimma University Ethical Review Committee. All information obtained is be kept anonymous. For this reason none of the personally identifiable information will not be used in the presentation of the findings in any form.

## Results

A total of 962 married women in the reproductive age group were included in the study giving the response rate of 100 %. The mean age of the women was 35(±6.8) years in the PHE Woreda and 34(±6.5) years in non-PHE Woreda. A total of 62.9 % of the women in the non-PHE Woredas and 69.8 % of them in PHE-Woredas had not gone to school or had attended informal education, while the rest had varying degrees of education (*P* < 0.001). Contrary to the finding on women, 36.5 and 45.4 % of the husbands in the PHE and non-PHE Woredas, respectively did not go to school or had attended informal education (*P* < 0.001). Regarding current school attendance, 50 % of the women in the PHE and 49 % of the women in the non-PHE Woredas were attending school at the time of the survey (*P* = 0.99). Mean family size was 6.2(±5.6) persons in the non-PHE Woredas; while it was 5.7(±4.1) in the PHE ones (*P* = 0.145).

The results showed that the percent of women who had heard about FP methods was similar between the PHE and non-PHE Woredas (92.5 and 95 %, respectively). There was no significant difference in the number of FP methods the women knew out of 13 total methods. However, when asked about the eight commonly used FP methods, women in the PHE Woredas mentioned 3.03(±1.64) methods while those in non-PHE Woredas knew 2.74(±2.26) methods (*P* = 0.032).

There was no significant difference in the CPR between the two woredas (70.7 % PHE vs 73.9 % non-PHE). However, the percent of new acceptors of modern FP methods was higher in the non-PHE Woreda (69.5 %) compared with the PHE Woredas (26.2 %, *P* < 0001). There was no significant difference in the percent of women who had heard of at least three contraceptive methods and in the percent of women who knew at least one source of modern FP methods between the two woredas.

When we examine the CPR by age category of women, there was a significant difference between the different age groups in both PHE and non-PHE Woredas showing that the CPR is lower in the younger age groups and in the older age groups.

As the CPR are very high in both woredas, we suspected that this could be due to the effect of Meskel holiday when there is a campaign on promoting family planning that has resulted in the culture of using family planning by the community. Meskel is a religious holiday commemorating the day on which the true Jesus’s cross was found. It is warmly celebrated in the month of September among Orthodox Christian in Ethiopia in general and in Gurage Zone in particular. In Gurage zone, usually husbands, who are mostly merchants in the towns, will come back to their rural wives. The Woreda Health Offices and health facilities target this holiday to distribute family planning methods. Therefore, we did subgroup analysis for married couples who are not new acceptors of modern family planning methods (those who are not acceptors of family planning for the first time during the last 1 year). As presented in Table 1, CPR was significantly (*P* < 0.0001) higher (78 %) in the PHE Woreda compared to non-PHE Woreda (52 %).

**Table 1:** Indicators FP outcomes by PHE and non-PHE Woredas

Variables	PHE (*n* = 480)	Non-HE (*n* = 482)	*P*
Heard about FP methods	92.9 %	95.0 %	*0.09900*
Mean number of FP methods known by women out of 13 different methods (±SD)	3.69(±2.73)	3.56(±2.76)	*0.49300*
Mean number of FP methods known by women out of out of eight commonly used FP methods (±SD) ^a^	3.03(±1.64)	2.74(±2.26)	*0.03200*
CPR	70.7 %	73.9 %	*0.34700*
CPR excluding new acceptors^*ƒ*^	78 %	52 %	*P < 0.0001*
Percent of new acceptors of modern FP methods	26.2	69.5	*P < 0.0001*
Percent of women in reproductive age who heard at least 3 contraceptives	67.5 %	65.5-0	*0.42800*
Percent of women who knew at least one source of modern contraceptives	96.1	96.7	*0.60800*
Percent of husband supporting use of FP methods himself	30.2 %	7.3 %	*P* < 0.0001

^a^The 8 commonly used family planning methods (Pills, Norplant, Depo-Provera, condom, IUCD, Tubal legation, vasectomy and Spermicids)

^ƒ^New acceptors are defined as women/couples who started using modern contraceptive methods for the first time during the last 1 year before the survey

Similarly, in the PHE Woredas, a larger percent (30.2 %) of men support the use of FP by themselves compared with the non-PHE Woredas (7.3 %), which is another striking finding (*P* < 0.001), Table 1.

**Fig. 3:**
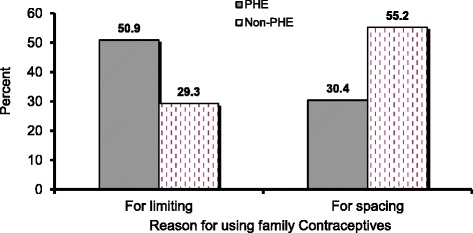
Differences in the purposes of using familiy planning among the current family planning users (*P *<0.0001)

Regarding the reason for FP use among the current users, more women in the PHE Woredas practiced FP for limiting their children (50.9 % vs. 29.3 %), while a larger proportion of women in the non-PHE Woredas used FP for spacing their children (55.2 % vs. 30.4 %), (Fig. 3).

As presented in Table 2, multivariable linear regression model showed after adjusting for different variables, women’s educational level, women’s occupation and husband’s occupation were significantly associated with knowledge of FP methods. Higher knowledge of FP methods was positively associated with increased years of education of the women (*P* < 0.0001), woman’s occupation of being a farmer (*P* = 0.005), women’s occupation of being a student/private employee (*P* = 0.001).

**Table 2:** Multivariable linear regression model predicting knowledge^a^ of family planning methods among married women of reproductive age groups in PHE and non-PHE Woredas

Predictors	ß	SE	*P*
Age of the women(years)	−0.009	0.014	*0.5180*
Women’s educational status	0.296	0.036	*<0.0001*
Husband’s educational status	0.017	0.017	*0.3300*
Woman’s occupation			
Housewife (Reference)			
Farmer	0.730	0.257	*0.0050*
Others	0.960	0.278	*0.0010*
Husband’s Occupation			
Farmer (reference)			
Employees	1.238	0.383	*0.0010*
Merchant	0.384	0.244	*0.1150*
Others	0.328	0.300	*0.2740*
Woreda			
Non-PHE(reference)			
PHE	0.171	0.181	*0.3450*

^**a**^Knowledge refers to number of family planning methods mentioned by women, *SE* standard error

We did sub-group analysis of women by excluding the new acceptors showed a similar finding regarding the predictors of knowledge of family planning. We also analyzed the predictors of current use of FP methods. On multivariable logistic regression model, after adjusting for different socio-demographic variables, older age of the women (*P* = 0.01) and husband’s desire for more children were negatively associated with current contraceptive use (0.032). On the contrary, husband’s support of FP use by his wife was very strongly associated with the current use of contraceptives (Table 3). Women whose husbands support their use of FP were 17 times more likely to use FP at the time of the survey (*P* < 0.0001).

**Table 3:** Multivariable logistic regression model predicting current use of family planning methods among married women of reproductive age groups in PHE and non-PHE Woredas

Predictors	AOR	95.0 % C.I		*P*
		Lower	Upper	
Age of the women	0.955	0.922	0.989	*0.0100*
Educational status of the women	0.997	0.919	1.083	*0.9510*
Educational status of the husband	0.995	0.960	1.032	*0.7920*
Total number of children the woman has	1.107	0.993	1.233	*0.0670*
Woman’s desire to have a child in the future	1.300	0.841	2.010	*0.2370*
Husband’s desire to have a child in the future	0.618	0.398	0.960	*0.03200*
Woreda				
Non-PHE	1			
PHE	0.933	0.632	1.377	*0.728*
Husband’s support of family planning use by his wife	17.219	11.079	26.762	*<0.0001*
Wealth Index Tertile				
Low	1.199	0.770	1.868	*0.4220*
Medium	0.773	0.492	1.216	*0.2650*
High				
Occupation of the woman	1			
House wife				
Farmer	1.690	0.956	2.988	*0.0710*
Others(employed by private or government, student)	0.722	0.407	1.281	*0.2660*
Occupation of the husband				
Farmer	1			
Employees(government or NGO)	2.003	0.677	5.926	*0.2090*
Merchant	1.019	0.603	1.725	*0.9430*
Others(employed by private, student)	0.604	0.326	1.119	*0.1090*

*AOR* adjusted odds ratio*, CI* confidence interval*, NGO* non governmental organization

A second multivariable logistic regression analyses using women who are non-new acceptors of family planning methods showed number of children that the woman has (*P* = 0.001), husband’s support of use of family planning methods by his wife (*P* < 0.0001) and living in the PHE Woreda (*P* < 0.0001) were strong positive predictors of current use of family planning methods. Women whose husbands support their use of family planning methods were over 20 times more likely to use family planning methods. Similarly, women in the PHE Woreda were over four times as likely to use family planning methods at the time of the survey (Table 4).

**Table 4:** Multivariable logistic regression model predicting current use of family planning methods among women who are non-new acceptors^a^

Predictors	AOR	95.0 % C.I.		
		Lower	Upper	*P*
Age of the woman	0.952	0.903	1.004	*0.068*
Educational status of the woman	0.934	0.813	1.074	*0.339*
Educational status of the husband	1.013	0.953	1.077	*0.686*
Desire for more children	1.664	0.849	3.262	*0.138*
Number of children the woman has	1.325	1.117	1.572	*0.001*
Husband’s desire for more children	0.702	0.349	1.415	*0.323*
Woman’s Occupation				
Housewife	1			
Farmer	2.350	1.084	5.098	*0.031*
Others(employed by private or government, student)	0.984	0.402	2.411	*0.972*
Occupation of the husband				
Farmer	1			
Employees	3.801	0.404	35.772	*0.243*
Merchant	1.061	0.445	2.527	*0.894*
Others(employed by private or government, student)	0.861	0.330	2.249	*0.761*
Woreda				
PHE	4.274	2.217	8.238	*P < 0.0001*
Non-PHE	1			
Husband’s support of contraceptive use by his wife				
Yes	20.352	9.708	42.665	*P < 0.0001*
No	1			
Household Wealth index Tertile				
Low	1			
Medium	1.598	0.667	3.830	*0.293*
High	1.869	0.744	4.695	*0.183*

^a^New acceptors are defined as women/couples who started using modern contraceptive methods for the first time during the last year before the survey

*AOR* adjusted odds ratio, *CI* confidence interval

Analyses of data from key informant interviews also indicated that family planning services are better integrated and decentralized into the grassroots level in the PHE Woreda (Table 5). It was observed that family planning services are linked to conservation activities with stronger use of schools as a medium for reaching parents. In-depth interviews with different sectors showed that, in the PHE Woreda, issue of family planning are integrated with health and environment. For instance, in giving health education, the issues of population are linked to the effect that they have on the environment and health. The Gurage People Self Development Program (GPSDO) program coordinator in the PHE Woreda stated:

**Table 5:** Summary of findings in-depth interview with key informants from different sectors

Issues	PHE Woreda	Non-PHE Woreda
Integration of Population, Health and environment issues during implementation	Family planning issues are better integrated with health and environmental issues at level of frontline workers of various sectors.	Networking of sectors at Woreda level in co-planning; however at the level of frontline workers of the different sectors interventions are vertical. There is no interaction of population health and environment issues
Main focus of interventions	Family planning, Reproductive Health (HIV and other communicable disease prevention and support of OVC) and environmental protection	Family planning and reproductive (HIV and other communicable disease prevention and support of OVC)
Income generating schemes	Are more diversified and customized to the environmental protection and food security eg. Beekeeping, banana plantation, Energy saving stove production, animal fattening, handcrafts, Corn farms of women’s groups. They are also targeted to women.	Limited although they are customized to environmental protection and targeted to women.
Information éducation & communication (IEC/BCC)	Integrated focusing on the interplay of population, health and environment.	Given vertically targeting mostly family palling with minimal/no focus on the inter play between population health and environment.
Strategies used for implementation	School clubs and students as a medium of reach out parents and as cadres of population health environment issues and community mobilization using Voluntary community Health workers(CBRHA)	More based on community mobilization using voluntary community health workers (CBRH agents).
Networking and communication between different sectors	PHE has a facilitation and capacity building role at Woreda and frontline level. Sectors have an implementation role. At the frontline workers level VCHWs have a mobilization role, while health extension (HEW) and development agents (DA) have an implementation role. They network very well at grassroots level.	GPSDO has a facilitation and capacity building role at Woreda and front line level. Sectors have an implementation role at Woreda level. At the frontline workers level VCHW have a mobilization role, while HEW and DA have an implementation role theoretically. But, in practice, networking is under built at the grassroots level. However, at the woreda level, the sectors have better network as they meet every month as Woreda Advisory Committee members (WAC).
A forestation activities	A total of 1,103,00 trees were planted during the previous fiscal year of which 75–80 % have survived	A total of 15,240 trees planted during the previous fiscal year. The status of survivors is not determined yet.
Percent of leadership positions held by the Natural resource management committee	10–15 %	10–15 %

“…*We have a philosophy of economics of scale in integrating the issues of population, health and environment. Students are involved in afforestaion and area closure activities in addition to passing the key messages to their parents. Rather than making different interventions at different times, integrating our intervention that addresses the issues of population, health and environment caters for the best use of resources. For example we have a very strong integrated school health program. We use school students as medium to educate their parents on family planning, health and environmental issues. We have school education program, students are educated by different sectors including health and agriculture on the link between population, health and environment. Health education is given twice per month in 5 schools. The other strategy is community mobilization where PHE has a capacity building and facilitation role.*”

There was also a diversified source of income generation mainly owned by women targeting environment and family planning. A significantly (*P* < 0.0001) higher proportion of households in the PHE Woreda (9.8 %) were using the energy saving stoves compared with those in the non-PHE Woredas (2.7 %). However, there was no difference in the percent of cooking places that have air vent (windows) between the PHE and non-PHE Woreda (Table 6).

**Table 6:** Use of energy saving stove and other environmental issues by Woreda

Variables	PHE (*n* = 480)	Non-HE (*n* = 482)	P
Household has separate kitchen	47.6 %	45.4 %	0.5010
The cooking place has window (air vent)	38.6 %	38.0 %	0.8390
Percent of women using energy saving stove	9.8 %	2.7 %	*P* < 0.0001
Average time spent to collect fire wood (hours)	2.03(±1.6)	2.17(±4.9)	0.5440

The qualitative data also indicated that 54 energy saving stoves were distributed both in the rural and urban areas of the in the PHE Woreda compared with non-PHE Woreda, where a total of 50 stove were distributed only in the urban areas. The stoves in the PHE Woreda were produced locally by organized women’s group, while the ones in the non-PHE Woreda were purchased from other areas. An in-depth interview with women using the energy saving stoves in the PHE Woreda indicated that in the energy saving is getting more acceptance.

One of the women using the energy saving stove stated the following to show how beneficial the stove is,

“…*we were about to sell our children in exchange for getting fuel, now as user of the energy saving stove, we are reaping the benefits. I am also one the women in involved in the production of the stove. We are very happy in producing as it is highly needed and accepted by the community*.”

The chair person of women‘s group working on energy saving stove production and income generating scheme in the PHE Woreda (Cheza Kebele) stated the following in trying to explain how their income generating scheme Operates:

“…*The project…GPSDO/PHE provided us a capacity building training on the production of energy saving stoves and assisted us with materials such constructing a house which we use for storing materials such as cement, metallic moulds and supplied us with inputs used for stove production. The kebele gave us a space to construct the house. Our group has now 28 members and we are now very skilled in producing the energy saving stoves and we are even training others. Initially, we use to sell a single stove for 23 Birr. Currently, the demand has increased and a single stove is sold for 200 Birr*.”

## Discussion

The findings showed that the PHE Woredas had better integrated PHE issues and networking at the community level. The fact that there was no significant difference in the CPR between PHE and non-PHE Woredas could be due to the effect of the Meskel campaign as the study was carried out right after the Meskel holiday. We did a subgroup analysis excluding the new acceptors of family planning to investigate if there is a difference among those women who were not new acceptors of family planning by avoiding the confounding effect of the Meskel holiday. The result showed a significantly (*P* < 0.0001) higher CPR in the PHE Woreda (78 %) compared to the non-PHE Woreda (52 %) indicating the effects of the positive behavioral changes that the PHE approach had introduced. Our findings also showed significant positive change in family planning behaviors in the PHE Woredas. Knowledge about FP methods that are commonly used was significantly higher in the PHE Woredas and larger proportion of husbands in the PHE Woreda supported the idea that husbands’ should use family planning methods. This finding is similar to the findings of a study in Philippines [6], which showed that an integrated model showed a better outcome compared with the single-sector models in terms of improvements in health, individual FP and reproductive health practices, and community-level indicators of food security and vulnerability to poverty.

The fact that a significantly higher proportion of couples used family planning methods for limiting the number of children in the PHE Woreda as compared to the non-PHE Woreda indicates the positive attitudinal change that the PHE approach has brought in building the norm of having smaller family size as the data also showed that desire for additional children is significantly lower in the PHE Woreda. The fact that the study was conducted immediately after the Meskel Holiday might have inflated the CPR in the both woredas.

Husbands’ support of their wives’ FP use was strongly associated with current use of FP methods. Studies in Ethiopia and Kenya also showed that communication between husband and wife on FP issues and husband’s support (approval) of FP use by his wife are critical factors determining FP outcomes including CPR and unmet need [13-14]. In this study, men in the PHE Woreda also support using FP methods themselves. These findings suggest the need for enhancing dialogue and advocacy to involve husbands in FP issues to improve FP outcomes.

The findings also showed that there is better integration of environmental conservation activities in the PHE Woredas into FP and health activities. For instance, there is better acceptance of fuel saving stoves, better afforestaion, and more diversified, environmentally friendly income generation activities in the PHE Woredas. This might be due to the positive attitude among community members as a result of the PHE-facilitated awareness creation activities using students as a medium and through community mobilization efforts by community based reproductive health agents (CBRHAs). Studies showed that the PHE approach empowers women and facilitates their involvement in the conservation activities [10] ensuring an effective and sustainable solution to population and environmental issues [6]. A report also indicated that despite the different challenges, PHE projects generate improved attitudes towards conservation and may play a critical role in laying the groundwork for successful conservation, particularly in areas where goodwill and trust in conservation organizations is not as strong [15].

Evidence shows that efforts to scale up programs in Madagascar and the Philippines has been relatively successful due to early and continued recognition of the interplay between FP and environmental issues by the conservation community and recognition by FP advocates and other health partners of the benefits of partnering with conservation organizations, among others [5, 6, 16, 17]. Efforts to address unmet need for FP in rural communities in Madagascar have been strongly influenced by local, regional, national and international FP, conservation, and development initiatives as well as through focused site-based PHE projects [18]. This implies that a very close partnership with the different sectors especially the natural resources management office is critical in ensuring the sustainability and scale up of the program.

With an ever increasing population and concomitant decline of natural resources, the findings highlight the effectiveness of PHE approach in promoting family planning outcomes in the rural communities of Ethiopia. This approach is critical and needs to be scaled up to address the problem food insecurity and malnutrition that is frequently encountering human population [19–22] especially in developing countries.

Although the study demonstrated significant differences between the PHE and non-PHE Woredas in terms of family planning behaviors, the following limitations need to be considered in interpreting the results. Due to the cross-sectional nature of the data, it was not possible to see the effect of improved family planning behaviors on the actual fertility practices. Due to lack of baseline population based data, it was not possible to show the effect of the PHE approach through the comparison of the difference of differences analysis. However, conscious effort has been made to minimize biases that could creep in by making the two woredas similar in terms of many of the known parameters that are relevant to the study. In fact the control woreda (Gumer) was closer to the main road which could have facilitated family planning outcomes in the control woreda. As the study was conducted during the Meskel Holiday, the family planning outcome measures were inflated in both woredas. Therefore, a subgroup analyses of the non-new acceptors was done to compare what has been happening during the last 5 years (2008–2012) before the survey when the project was in operation.

## Conclusions

Based on the findings from the different data sources, the following are outstanding observations:

1. Women in the PHE Woredas had a significantly better knowledge of commonly used FP methods compared with those in the PHE Woreda.
2. There is better integration of FP, health and environmental issues into the grassroots level interventions in the PHE Woreda.
3. Women in PHE Woreda were more likely to use family planning methods after adjusting for other factors when new acceptors were excluded to avoid the effect of the Meskel Holiday.
4. Husband’s support of family planning use by their wives is also strong independent predictor of family planning use among married women.
5. The PHE approach leads to value added outcomes for family planning behaviors such as a significantly higher number of men supporting FP use by themselves, showing that the approach is stronger in enhancing the involvement of men in family planning services and production and use of fuel saving stoves.

### Recommendations

Based on the above findings, the following key actions are recommended for further improvement of the family planning outcomes

1. Strategies used in the PHE Woredas such as using schools and students as medium for integrated PHE interventions are commendable approaches that need to be strengthened.
2. Future interventions on family planning need to target husbands (men) to enhance family planning use.
3. Integration of population, health and environmental issues needs to be strengthened and scaled up to sustain the positive FP behaviors such as support of FP use by the husbands.
4. More networking and integration with key stakeholders such Woreda Environmental Conservation office in the Woreda Agriculture Office is critical to enhance ownership and sustainability of the program.
5. The PHE approach should be scaled up to more woredas as far as possible.
6. Future research should evaluate the effect of better family pinning behaviors among married women and their husbands in the PHE Woreda on TFR.
